# Mitochondrial phylogeography of baboons (*Papio *spp.) – Indication for introgressive hybridization?

**DOI:** 10.1186/1471-2148-9-83

**Published:** 2009-04-23

**Authors:** Dietmar Zinner, Linn F Groeneveld, Christina Keller, Christian Roos

**Affiliations:** 1 Cognitive Ethology, Deutsches Primatenzentrum, Kellnerweg 4, D-37077 Göttingen, Germany; 2 Behavioral Ecology and Sociobiology, Deutsches Primatenzentrum, Kellnerweg 4, D-37077 Göttingen, Germany; 3 Institute of Farm Animal Genetics, Friedrich-Loeffler-Institut, Neustadt, Germany; 4 Göttinger Zentrum für Biodiversitätsforschung und Ökologie, Untere Karspüle 2, D-37073 Göttingen, Germany; 5 Gene Bank of Primates and Primate Genetics, Deutsches Primatenzentrum, Kellnerweg 4, D-37077 Göttingen, Germany

## Abstract

**Background:**

Baboons of the genus *Papio *are distributed over wide ranges of Africa and even colonized parts of the Arabian Peninsula. Traditionally, five phenotypically distinct species are recognized, but recent molecular studies were not able to resolve their phylogenetic relationships. Moreover, these studies revealed para- and polyphyletic (hereafter paraphyletic) mitochondrial clades for baboons from eastern Africa, and it was hypothesized that introgressive hybridization might have contributed substantially to their evolutionary history. To further elucidate the phylogenetic relationships among baboons, we extended earlier studies by analysing the complete mitochondrial cytochrome *b *gene and the 'Brown region' from 67 specimens collected at 53 sites, which represent all species and which cover most of the baboons' range.

**Results:**

Based on phylogenetic tree reconstructions seven well supported major haplogroups were detected, which reflect geographic populations and discordance between mitochondrial phylogeny and baboon morphology. Our divergence age estimates indicate an initial separation into southern and northern baboon clades 2.09 (1.54–2.71) million years ago (mya). We found deep divergences between haplogroups within several species (~2 mya, northern and southern yellow baboons, western and eastern olive baboons and northern and southern chacma baboons), but also recent divergence ages among species (< 0.7 mya, yellow, olive and hamadryas baboons in eastern Africa).

**Conclusion:**

Our study confirms earlier findings for eastern Africa, but shows that baboon species from other parts of the continent are also mitochondrially paraphyletic. The phylogenetic patterns suggest a complex evolutionary history with multiple phases of isolation and reconnection of populations. Most likely all these biogeographic events were triggered by multiple cycles of expansion and retreat of savannah biomes during Pleistocene glacial and inter-glacial periods. During contact phases of populations reticulate events (i.e. introgressive hybridization) were highly likely, similar to ongoing hybridization, which is observed between East African baboon populations. Defining the extent of the introgressive hybridization will require further molecular studies that incorporate additional sampling sites and nuclear loci.

## Background

**Figure 1:**
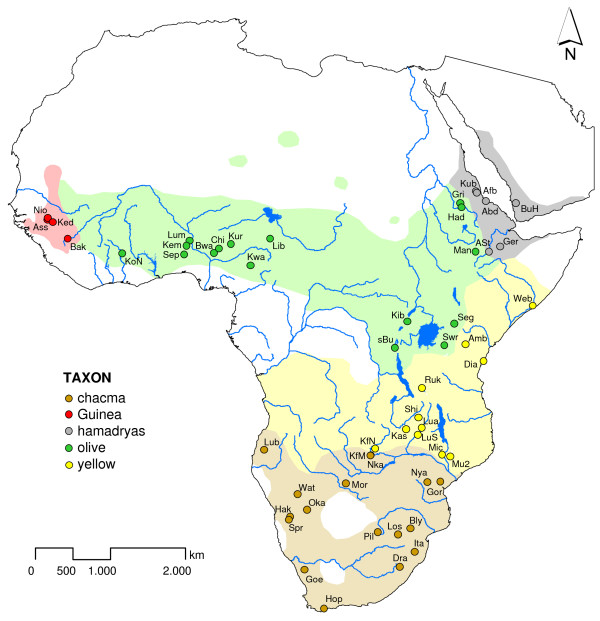
**Geographical distribution of baboons**. Distribution based on the map in Kingdon [11], revised in accordance with Sarmiento [12] and Galat-Luong et al. [13]. Sampling sites are indicated by coloured points and labelled with a three-letter code according to Additional file 1.

The contribution of reticulate evolution, in particular natural and introgressive hybridization to the origin and development of humans and primates in general has recently gained much attention [1-7]. One primate species complex that turned out to be of particular interest in this respect are baboons (*Papio *spp.). They diverged on a time scale analogous to that of *Homo *in similar, if not the same habitats in southern and eastern Africa, and it has been proposed to use them as an analogous model, when exploring the evolution of the hominin lineages [8,9]. It was estimated that they have radiated across Africa approximately 1.8 million years ago (mya) [10]. Baboons are now distributed all over sub-Saharan Africa, excluding most parts of the west and central African rainforest, and have even colonized parts of the Arabian Peninsula [11-13] (Figure 1). They form morphologically and geographically distinct populations but demonstrate no pre- or postzygotic reproductive isolation [8,14-16]. Additionally, baboons are known to produce fertile hybrid offspring with geladas (*Theropithecus gelada*) [17,18]. The divergence between these sister genera is estimated at 3.5–4 mya [[19,20] and references therein]. The five generally acknowledged baboon types are the Guinea baboon (*P. (hamadryas) papio*) from West Africa, the olive baboon (*P. (h.) anubis*) from the northern savannah belt, the hamadryas baboon (*P. (h.) hamadryas*) from north-east Africa and the south-western Arabian Peninsula, the yellow baboon (*P. (h.) cynocephalus*) from the East African coastal lowlands, Zambia and Angola and the chacma baboon (*P. (h.) ursinus*) from southern Africa (Figure 1).

The taxonomic status of the five baboon types has been debated for more than 50 years without reaching a consensus [for reviews see [21,22]]. They are either classified as (allopatric) subspecies of the superspecies *Papio hamadryas *[22,23] or as distinct species [24-26]. In addition to the five traditional types several other geographic variants have been described [21], of which at least some deserve the same taxonomic level as the five basic types [8,22,23]. Here we follow Groves [24], Grubb et al. [25] and Jolly [26] and recognize baboon allotaxa as phylogenetic species. Since the question regarding the appropriate taxonomic level is more a matter of philosophy, depending largely on the underlying species concept [27], an accurate and well-supported phylogeny is crucial for understanding the evolution of the genus. This is of particular relevance for all studies where phylogenies have to be correlated such as in co-evolution studies, for example, the evolution of primates and their pathogens [28]. Similarly, in studies concerning the evolution of certain traits, such as particular heritable social behaviours, it is important to distinguish between ancestral (plesiomorphic) and derived (apomorphic) states. If, for example, hamadryas baboons are the most basal baboons, as in the phylogeny proposed by Purvis [29], their peculiar social organization with its one-male units [30] could be regarded as the ancestral social organization of the baboon clade and not the multi-male organization of other baboon taxa [31]. Hence, incorrect phylogenies will inevitably lead to incorrect inferences about the evolution of species.

The origin of *Papio *is suggested to be in southern Africa [fossil evidence: [32-34]] and the oldest mitochondrial haplotypes so far were found in chacma baboons which live in southern Africa [10]. Apart from its origin, the evolutionary course of different baboon taxa is a subject of great debate and speculation. Based on morphological and behavioural characters several contradictory intra-generic phylogenetic hypotheses have been proposed [for a review see [10]]. In molecular studies discordance between mitochondrial DNA (mtDNA) phylogenies and morphology-based taxonomy became obvious, suggesting that reticulation events affected the evolutionary history of baboons [10,15,35,36].

In this study, we determined the phylogenetic relationships within the genus *Papio *and estimated the dates of divergence events between lineages. Our analyses are based on complete mitochondrial cytochrome *b *gene (cytb) and 'Brown region' [37] sequences. We expanded geographic and taxon sampling to make our phylogenetic analysis more robust [38], because taxon sampling is particularly important in paraphyletic species [38,39].

Thus, our sampling covered most of the range of *Papio *and included previously unsampled regions and taxa, and does not include samples from captive animals with the exception of one sample from a confiscated animal in Cameroon. Therefore only samples of clear provenance are present in the analysed data set. This enabled us to extend previous analyses substantially and to refine the phylogenetic resolution among mtDNA haplotypes. Comparisons of the biogeographic distribution of mtDNA haplotypes and morphotypes allowed us to detect indications for introgressive hybridization and reticulate evolution.

## Results

### Phylogenetic relationships

**Figure 2:**
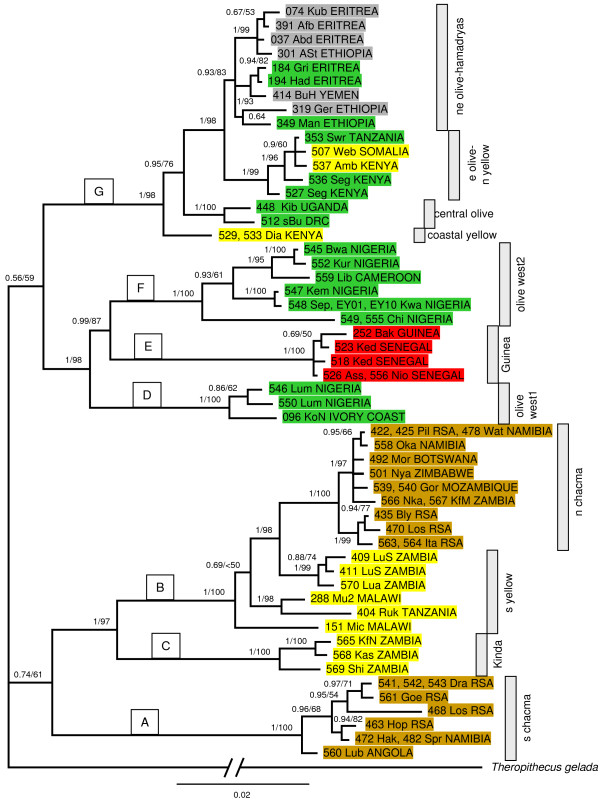
**MtDNA phylogeny of baboons**. Bayesian phylogram based on combined 'Brown region' and cytb haplotype sequences from 67 *Papio *individuals. Tip labels contain the unique identifier (number), the sampling site in form of a three-letter code as depicted in Figure 1 and country of origin of sequences within a haplotype. Colours indicate the traditionally recognized baboon species: brown = chacma, red = Guinea, grey = hamadryas, green = olive and yellow = yellow. Bayesian posterior probabilities and maximum-likelihood bootstrap values are depicted above the branches. A – G indicate the seven major clades, grey bars on the right indicate ten terminal haplogroups and the paraphyletic clade of southern yellow baboons.

We successfully amplified and sequenced the mitochondrial 'Brown region' and the complete cytb gene from 67 individuals from 53 localities (Additional file 1). Concatenated 'Brown region' and cytb sequences (2,036 bp) resulted in 54 haplotypes (excluding outgroup), which were defined by 435 variable sites, of which 338 were parsimony-informative. Identical haplotypes were found mainly in animals, that came from the same locality (same social group), but were also found from sites located more than 1,100 km apart (Figures 1, 2; Additional file 1). When examining each locus separately, 47 haplotypes were detected in the 'Brown region' (896 bp), defined by 182 variable sites, of which 140 were parsimony-informative. In baboons from the southern part of the Republic of South Africa (RSA), southern and western Namibia and western Angola, a triplet deletion in the ND5 gene of the 'Brown region' was detected. The complete cytb sequence (1,140 bp) contained 252 variable sites, of which 198 were parsimony-informative defining 50 haplotypes.

According to the Akaike Information Criterion (AIC), the 'Brown region' was found to best fit the Tamura-Nei model (TrN), while for both the cytb gene alone and the concatenated data set a Transition model (TIM) was selected. For all data sets a model with both gamma distributed rate heterogeneity (Γ) and a proportion of invariant sites (I) was proposed.

Simple neighbor-joining reconstructions based on uncorrected distances for both data sets (cytb and 'Brown region') revealed identical tree topologies and similar branch lengths (data not shown), indicating that both data sets contain solely mitochondrial fragments and no nuclear pseudogenes. Moreover, no inconsistent nucleotides were detected in overlapping regions of both studied loci.

Detailed phylogenetic relationships were estimated with Bayesian and maximum-likelihood (ML; -lnL = 7359.8389 – 7360.1104) approaches, which both led to identical topologies and similar support values (Figure 2). Several well-supported clades were detected which however, do not concur with the five baboon species. The only exception are Guinea baboons, which form a monophyletic group. All other species were mitochondrially paraphyletic. In contrast, we found a strong geographical signal with local populations forming monophyletic groups irrespective of their species affiliations, which clearly showed the discordance between mtDNA phylogeny and morphology (Figure 2).

In total, seven larger haplogroups were detected (clade A-G; Figure 2), which are distributed in two major clades, a southern and a northern one. However, respective monophylies of the southern and northern clades are only weakly supported, suggesting that the divergence of the two clades most likely happened within a short time period. The major southern clade further divides into three clades, the southern chacma clade (A), representing haplotypes from the Drakensberg, the Cape, western RSA, southern and western Namibia and south-western Angola, the northern mixed chacma and yellow baboon clade (B), which includes chacma haplotypes from Mozambique, northern RSA, Zimbabwe, Botswana, south-central Zambia and north-eastern Namibia, and yellow baboon haplotypes from Malawi, eastern Zambia and south-eastern Tanzania, and finally, (C) a clade of baboons that originated from central Zambia, which morphologically represent Kinda baboons. The triplet deletion in the ND5 region is an autapomorphy of clade (A) since it was not found in any other papionin.

The major northern clade divides into a western and an eastern clade. The western clade consists of one clade of olive baboon haplotypes found in Ivory Coast and western Nigeria (D, west1), a second clade of olive baboon haplotypes mainly from central and eastern Nigeria and northern Cameroon (F, west2), and a well-defined clade of Guinea baboon haplotypes covering the area from Guinea to Senegal (E). The eastern clade is comprised of a mixture of different species. The first separation concerns yellow baboons from the Kenyan coast, whereas the second includes olive baboons (central olives) from western Uganda and eastern Democratic Republic of Congo (DRC). Furthermore, we found two more clades, one comprised of yellow and olive baboon haplotypes from northern Tanzania, Kenya and Somalia while the other represents a mixture of olive and hamadryas baboon haplotypes from Ethiopia, Eritrea and the Arabian Peninsula.

**Figure 3:**
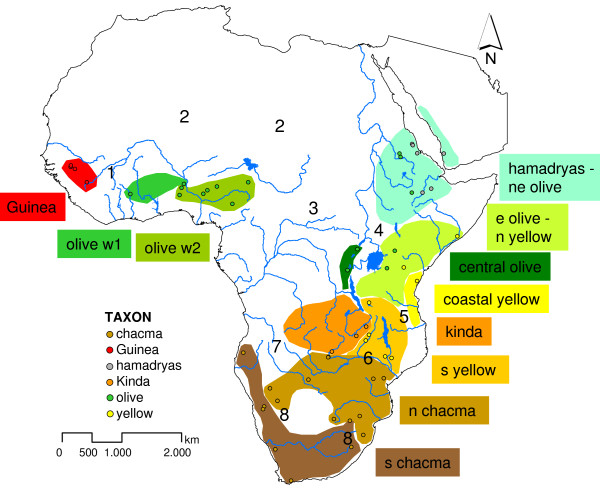
**Geographical distribution of mtDNA-haplogroups**. Approximate geographical distribution of terminal haplogroups. Points represent sample locations, shaded areas approximate ranges of haplogroups. Numbers indicate important future sampling areas (1 = contact zone between olive and Guinea baboons in Mauretania, 2 = isolated olive baboon populations from the Sahara desert, 3 = olive baboons from the Central African Republic, Congo and north eastern DRC, 4 = contact zone between olive-hamadryas, olive-yellow and central olive baboon clades, 5 = contact zones between coastal, northern and southern yellow baboons, 6 = contact zones between Kinda, northern chacma and southern yellow baboons, 7 = baboon populations in eastern Zambia and Angola, 8 = contact zones of southern and northern chacma baboons).

In total, ten well-supported terminal clades/lineages became apparent in our study, which represent geographic populations or demes. The only exceptions are southern yellow baboons, which are paraphyletic. The approximate geographical distribution of the respective haplogroups is shown in Figure 3.

### Estimation of divergence times

The data set used to estimate divergence times contains 2,023 bp of sequence information from 27 primate individuals. Over half of the characters (1,071 bp) were variable and 787 were parsimony-informative. The coding regions were found to best fit a TrN model with both gamma distributed rate heterogeneity (Γ) and a proportion of invariant sites (I), and non-coding regions were found to best fit a TVM + Γ + I model.

**Figure 4:**
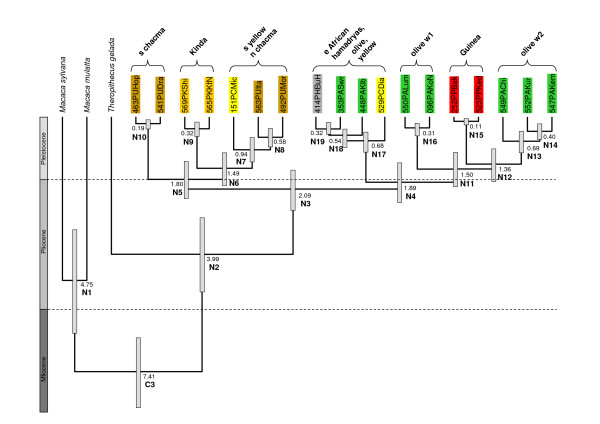
**Divergence age estimates**. Ultrametric tree with divergence age estimates resulting from the combined posterior distribution of 45,002 samples from four replicates of the BEAST analysis based on combined 'Brown region' and cytb sequence data from 18 *Papio *individuals. The mean age estimate for each node is given in mya, with the respective 95% credibility intervals indicated by the grey bars. Nodes of interest are arbitrarily numbered (N1-N19). C3 refers to one of the three nodes used for calibration (C1 and C2 not shown). A geological time scale is given on the left. Full details of age estimates are presented in Table 1.

Using three divergence dates as calibrations (C1: *Pan*-*Homo*, C2: *Pongo*-African apes and C3: *Macaca*-*Papio *in Table 1 and Figure 4), the *Papio*-*Theropithecus *divergence (N2) was dated at 3.99 million years ago (mya) (95% confidence limits [CI]: 2.92–5.09 mya), which is in the same range as both, the estimate given by Newman et al. [10] and the divergence date suggested by fossil evidence (3.5–4.0 mya) [[19,20] and references therein]. The subsequent divergences within *Papio *are considerably younger (~2 mya) and started in the Late Pliocene. The divergence of the ancestral *Papio *stem into a southern and a northern lineage (N3) was inferred to have occurred at 2.09 (1.54–2.71) mya. Shortly afterwards, the respective northern lineage diverged further into a western and an eastern clade (N4, 1.89 [1.33–2.48] mya) and the respective southern lineage into a southern and a northern proto-chacma baboon clade (N5, 1.80 [1.28–2.36] mya). Within the lineage leading to northern chacma baboons, Kinda baboons diverged 1.49 (1.03–1.98) mya (N6), followed by the separation of southern yellow baboons 0.94 (0.58–1.30) mya (N7). The age of the most recent common ancestor (MRCA) of the north-western lineage leading to Guinea and western olive baboons was estimated at 1.50 (1.02–2.02) mya (N11). In this lineage, the earliest divergence was between olive baboons of clade w1 and a common clade of Guinea/olive w2. The separation between the latter was estimated at 1.36 (0.91–1.86) mya (N12).

**Table 1:** Bayesian divergence date estimates in mya. Means and 95% credibility intervals are given for 25 nodes (see also Figure 3).

Node		mean	95% credibility interval
C1*	*Homo*/*Pan*	6.43	5.85–7.01
C2*	Homininae/Ponginae	13.74	12.59–14.90
C3	*Macaca*/*Papio*	7.41	6.42–8.46
N*	Hominoidea/Cercopithecoidea	24.38	18.98–30.33
N*	Colobinae/Cercopithecinae	15.63	11.50–20.08
N*	Cercopithecini/Papionini	9.80	7.72–12.07
N1	*M. sylvanus/M. mulatta*	4.75	3.27–6.29
N2	*Theropithecus*/*Papio*	3.99	2.92–5.09
N3	*Papio*	2.09	1.54–2.71
N4	northern clade	1.89	1.33–2.48
N5	southern clade	1.80	1.28–2.36
N6	n chacma + s yellow/Kinda	1.49	1.03–1.98
N7	n chacma/s yellow	0.94	0.58–1.30
N8	n chacma	0.58	0.32–0.86
N9	Kinda	0.32	0.14–0.55
N10	s chacma	0.19	0.06–0.33
N11	olive w2 + Guinea/olive w1	1.50	1.02–2.02
N12	olive w2/Guinea	1.36	0.91–1.86
N13	olive w2	0.69	0.41–1.03
N14	Kur/Kem	0.40	0.18–0.64
N15	Guinea	0.11	0.03–0.22
N16	olive w1	0.31	0.12–0.53
N17	coastal yellow – n yellow + central olive + e olive + ne olive + hamadryas	0.68	0.40–0.97
N18	central olive – n yellow + e olive + ne olive + hamadryas	0.54	0.32–0.78
N19	e olive + n yellow – ne olive + hamadryas	0.32	0.16–0.51

* not shown in Figure 4

Nodes used as calibrations are labelled with a "C", all others with an "N".

In the north-eastern clade, coastal yellow baboons diverged first 0.68 (0.40–0.97) mya (N17), followed by central olive baboons 0.54 (0.32–0.78) mya (N18). Eastern olive and northern yellow were mitochondrially separated from north-eastern olive and hamadryas baboons 0.32 (0.16–0.51) mya (N19). The divergence ages for the north-eastern clade fall in the same range as found in earlier studies. Newman et al. [10] estimated separations within the north-eastern clade of less than 0.62 (0.58–0.66) mya. Shotake et al. [40] reported that Ethiopian olive and hamadryas baboons have been separate lineages since approximately 0.34 mya.

## Discussion

Our phylogenetic reconstruction suggests that baboon populations can be diagnosed through mtDNA and are sorted into several coherent and reasonably well-supported haplogroups that do not match with recognized baboon species. The discordance between mitochondrial phylogeny and baboon morphology, which is most likely equivalent to a discordance between mitochondrial and nuclear phylogeny, results in mitochondrial paraphyly, which is common among animal taxa including primates [36,41-44].

The observed discordance might be explained by introgressive hybridization or incomplete lineage sorting since both can result in similar phylogenetic patterns and hence, complicate interpretation of phylogenetic reconstructions [45,46]. Although we can not rule out completely that incomplete lineage sorting may have had an effect, the strict geographical pattern of the baboon haplogroups provides some evidence against incomplete lineage sorting, because lineage sorting is a random process and the paraphyletic relationships that result from the failure of haplotypes to sort during speciation events should be random with respect to geography [47]. In contrast, in our mitochondrial phylogeny geographic close populations cluster together, which is often a strong indication of reticulation [41]. Thus, we conclude that introgressive hybridization rather than incomplete lineage sorting has resulted in the discordance between mitochondrial and morphological phylogeny present in baboons.

The introgression hypothesis becomes even more likely by the fact that baboons and other papionins show a strong tendency for intra- and intergeneric hybridization. Generic hybridization was observed between *Papio *and *Theropithecus *and between *Papio *and *Rungwecebus *[17,18,48]. For baboons, intrageneric hybrid zones are known for olive and northern yellow baboons [10,16,49-51], olive and hamadryas baboons [35,36,40,52] and Kinda and southern yellow baboons [15].

Male dispersal and female philopatry are the norm in baboons and other papionins [53] and one can assume that this is the ancestral state. Therefore male introgression would be the most likely introgression scenario, where males from one taxon invade groups of a neighbouring taxon and reproduce successfully. Extensive backcrossing of the hybrid offspring over generations with more invading males of the first taxon would result in nuclear swamping. This process can lead to the extinction of the introgressed taxon and only their mitochondrial genomes would remain as vestiges of their former existence in a population which morphologically would be the same as the introgressing taxon.

Together with a recurrent isolation and reconnection of local baboon populations in many parts of their range due to climate changes the male introgression and nuclear swamping hypothesis can explain the striking discordance between mitochondrial phylogeny and baboon morphology.

### Phylogeography

The paraphyletic pattern suggests a complicated biogeographic history of baboon species. Multiple phases of isolation, hybridization and introgression are highly probable, most likely triggered by the multiple cycles of expansion and retreat of savannah biomes during Pleistocene glacial and inter-glacial periods [54-56]. During the last 2.5 million years there have been about 20 glacial cycles of major forest expansions and retreats in Africa [57,58]. Environments during glacial periods were characterized by dry, tropical scrub and grassland with limited gallery forest along drainages, and thus, with the retreat of dense forest into high altitude and large river refugia, savannah corridors opened and provided pioneering and recurrent dispersal possibilities for savannah adapted mammal populations, such as baboons. Evidence for the multiple savannah and forest cycles come from pollen core data and the current distribution patterns of forest organisms [58-63]. The divergence of the *Papio *lineage accompanied by the dispersal from southern Africa to the north (~2.1 mya) and the further division into several distinct southern (~1.8 mya) and northern lineages (~1.9 mya) appears to be temporally in accordance with the expansion of savannah habitats and a major radiation of antelopes (Bovidae) and the diversification of hominins [64], all as a result of the onset and recurrence of the northern hemisphere glaciations since ~2.5 mya.

A similar phylogeographic pattern of an early north-south division and the subsequent division of the northern clade into an eastern and western clade have also been shown for other large African savannah mammals, such as hartebeest *Alcelaphus buselaphus *[65,66], topi *Damaliscus lunatus* and wildebeest *Connochaetes taurinus*[65], roan antelope *Hippotragus equinus *[67], warthog *Phacochoerus africanus *[68], giraffe *Giraffa camelopardalis *[69] and lion *Panthera leo *[70]. Within several of these taxa paraphyletic relationships were detected, similar to the pattern found in baboons [65]. These biogeographic patterns have been interpreted as results of Plio-Pleistocene climate cycles when the expansion of savannah habitats supported the dispersal of these taxa, although not for all taxa on the same time scale. However, a detailed analysis of these processes and a comparison with detailed data on climate and habitat change in Africa during the last 2.5 mya is far beyond the scope of our paper.

Our findings are almost consistent with the scenario for the mitochondrial population structure of *Papio *laid out by C.J. Jolly in his "North-south Split" and "Philopatry at the Frontier" hypotheses [C.J. Jolly pers. comm., [22,71]]. From our data the following scenario can be developed. Baboons dispersed to the south and north from a southern African origin at times when climate and habitat conditions were suitable (~2.1 mya), that is if during dry conditions dense forests were replaced by savannah and savannah woodland. There is evidence from various sources that two savannah corridors existed during arid periods in the Plio-Pleistocene, which baboons could have followed on their way north [58,72-75]. One migration route lead through a savannah corridor in eastern Africa and a second through a savannah corridor in eastern DRC west of the Pleistocene forest refuge in the Rift Mountains.

Baboons migrating to the north subsequently dispersed over the complete northern savannah belt, from Eritrea to Senegal. Changes of the climate to more humid condition with expanding forests probably led to the isolation of local baboon populations, because for most baboons dense forests constitute biogeographical barriers. These allopatric populations evolved for certain periods of time independently. Subsequent climate changes to more arid conditions again opened savannah corridors so that isolated populations were able to reconnect, with the chance of gene flow. As an extreme outcome, some of the historical baboon populations might have been "swallowed" by invading populations and became extinct. Male olive baboons, for example, reproduced with females from neighbouring populations, with subsequent nuclear swamping. Olive baboons, thus, most likely absorbed these local baboon forms, such as w1 and w2, from which we can still find the mitochondrial genome (clade 4 and 5) in western olive baboons. A similar process occurred in east and north-east Africa where olive baboons from Kenya and northern Tanzania carry mitochondria derived from a neighbouring yellow or proto-yellow baboon stock or in Ethiopia and Eritrea, where they carry hamadryas or proto-hamadryas mitochondria. In the latter cases, parts of the original populations still exist, as northern yellow and hamadryas baboons. However, hybridization between olive and yellow baboons and olive and hamadryas baboons (hybrid zones in Awash, Ethiopia [14] and Amboseli, Kenya [16]) is still going on. It is also likely that hybridization occurs at the western end of the olive baboon range, where olive and Guinea baboons meet. Similarly, introgression and nuclear swamping can explain why yellow baboons of Zambia carry mitochondria only distantly related to those of northern Tanzanian and Kenyan yellow baboons, and chacma baboons from Botswana and Zambia are mitochondrially very distant from those living in South Africa. In case of southern and northern yellow baboons a possible scenario could be that yellow baboons introgressed from the south into a neighbouring northern population (possibly proto-hamadryas) after the retreat of an East African forest barrier. Complete nuclear swamping would lead to a replacement of the local northern population with yellow baboons, which, however, would carry the mitochondria of the northern population. In case of chacma baboons we assume that they introgressed into parts of the southern yellow baboon population, thus replaced the yellow baboon nuclear genome, but retained the yellow baboon mitochondria. These events most likely took place early in the speciation process so that there was time for resulting chacma baboon population to evolve further into grey-footed chacmas. Interestingly, Zinner et al. [48] found that the recently described genus *Rungwecebus *[76,77] also carries mitochondria of the northern chacma – southern yellow baboon haplogroup, suggesting that also on this intergeneric level introgression events occurred. Our introgression hypothesis is partly built on similar sexually-differentiated introgression scenarios envisioned by Wildman et al. [36].

### Taxonomic implications

Our study shows that mtDNA does not constitute a useful marker for the identification of the five traditionally recognized *Papio *species. If terminal clades in our phylogeny would be regarded as phylogenetic species, one would have to split haplogroups of the same morphotype into different species (e.g. various olive baboon haplogroups, which most likely are not only similar in their morphology but also in their behaviour and ecology) or one would have to lump members of different morphotypes as one species, e.g. hamadryas and eastern olive baboons which are different not only in their morphology but even more in their social system [14,78,79]. Guinea baboons, which morphologically and genetically constitute a monophyletic clade seem to be the only exception. However, there are indications for introgression also from western olive baboons into Guinea baboons [80].

Despite these limitations, our data, in combination with morphological [23,81], behavioural [82] and additional genetic data [15] support the recognition of Kinda baboons as a distinct species (*P. kindae*). In our sample, we did not find any overlap between Kinda and either southern yellow or northern chacma baboons. In contrast, in a more detailed study of the Kinda baboons and neighbouring baboon populations, Burrell [15] found some evidence for gene flow between Kinda and yellow baboons in a narrow contact zone in eastern Zambia. Similarly, Clifford Jolly (pers. comm.) found some intermediate morphotypes between grey-footed and Kinda baboons where they meet.

Baboons of the southern chacma haplogroup possibly represent Cape chacmas (*P. (u.) ursinus*) and those of the northern chacma haplogroup grey-footed chacmas (*P. (u.). griseipes*), as was proposed by Jolly [8,22]. However, it remains to be explored, whether various haplogroups of yellow and olive baboons from eastern and western Africa may represent formerly described, but now synonymised taxa [21,83].

## Conclusion

The present study shows paraphyly in the genus *Papio *and discordance between baboon morphology and mitochondrial phylogeny. Accordingly, a classification of baboons based on mitochondrial sequence data is inappropriate. However, the geographic distribution of haplogroups provides insights into the evolutionary history of baboons and emphasizes the possible role of introgressive hybridization. Periodical isolation of populations and subsequent range overlap, most likely triggered by climatic changes, might have led to a gradual hybridization influencing baboon diversification and speciation [6]. The extent of introgression reaches from ongoing introgression of male olive baboon into yellow baboon population in Amboseli to the complete extinction of historical populations or taxa in west or south-east Africa by introgression where only the former mitochondrial genome remained as a trace in the current olive or grey-footed chacma baboon populations. Since the evolutionary history of baboons shows some similarities with that of the human lineage, it seems plausible that the introgression scenario depicted herein might also be an extended model for reconstructing the evolution of *Homo*.

Defining the extent of the reticulate (i.e. introgressive hybridization) events will require further molecular studies that incorporate additional samples and in particular nuclear loci. Our study showed that collecting samples from all over the baboon range with known provenances is indispensable, but samples from further locations as indicated in Figure 3, should be included in ongoing studies. The analysis of nuclear loci, however, might be hampered by the fact that nuclear sequence variation is expected to be relatively low in baboons as shown for parts of the Y-chromosome [84].

## Methods

### Sample collection

Faecal material from 64 individuals representing all five *Papio *species was collected from free ranging populations at 51 sites in Africa and the Arabian Peninsula between 1995 and 2007. One additional sample (No. 559) was provided by the Limbe Wildlife Centre, Cameroon. Although this specimen originated from northern Cameroon, its exact provenance is not traceable. Two other samples consisted of dry tissue from museum specimens (No. 404: *Papio cynocephalus*, north-east bank of Lake Rukwa, Tanzania, coll. no. 03-74959 Humboldt Museum, Berlin, Germany) or of tissue preserved in ethanol (No. 507: *Papio *(*ruhei*) *cynocephalus*, 40 km NW of Mogadishu at Webi Shebelli, Somalia, Zoologische Staatssammlung München, Germany). To obtain adequate geographical sampling, our sampling pattern covered most of the baboon range (Figure 1) with one major gap between Cameroon and Ethiopia, an area where only one baboon taxon is known to occur (*Papio a. anubis*) [21]. The geographic coordinates of the sampling sites were determined with GPS or, in the case of the two museum specimens, estimated from maps (Additional file 1). One *Theropithecus gelada *faecal sample was collected from a zoo animal (Zoo Duisburg, Germany). Either fresh or dry faecal material was collected. Fresh samples were preserved in 75% ethanol and dry samples in plastic tubes without any additive. Samples were stored at ambient temperature for up to six months before further processing.

### DNA extraction, PCR amplification and sequencing

DNA from tissue and faecal material was extracted using the DNeasy Blood & Tissue or QiAamp DNA Stool Mini Kits from Qiagen. To prevent contaminations, laboratory procedures followed described standard protocols [44,85-87]. DNA extraction, PCR, gel extraction and sequencing were performed in separate laboratories and repeated randomly after several months, in which case only one individual per species or location was tested. All PCR reactions were performed with negative (HPLC-purified water) controls.

We amplified and sequenced two parts of the mtDNA genome, 1) the 'Brown region' [88], which, in baboons, comprises 457 bp of the 3' end of the NADH dehydrogenase subunit IV (ND4) gene, the tRNA genes for histidine (His), serine (Ser), and leucine (Leu), and 239 of the 5' end of the NADH dehydrogenase subunit V (ND5) gene [36], and 2) the complete cytb gene.

The 'Brown region' (896 bp) was amplified via two overlapping fragments according to PCR conditions and primers as described in Newman et al. [10]. The complete cytb gene (1,140 bp) was also amplified via two overlapping PCR products, each with a size of 600–700 bp. To exclude miss-amplification of nuclear pseudogenes, primers were selected which amplify mitochondrial fragments only in papionins and not in any other primate group. The 5' end portion was generated with the oligonucleotide primers cytb1F (5'-GATACGAAAAACCATCGCTGT-3') and cytb1R (5'-AGTAGGGATGGAAGGTGATTT-3'). For the 3' end portion, the two primer pairs cytb2aF (5'-TTCGGCATCGTCACCCTCAC-3')/cytb2aR (5'-ATTTCTGGTTTACAAGACCAGT-3') and cytb2F (5'-CCTACCATTCGGCATCGTCG-3')/cytb2R (5'-GCTCCATTTCTGGTTTACAAG-3') were used to amplify the fragment in southern chacma baboons and in all other papionins, respectively. PCR conditions for all cytb amplifications were identical and comprised a pre-denaturation step at 94°C for 2 min, followed by 40 cycles at 94°C for 1 min, 60°C for 1 min and 72°C for 1 min, and a final extension step at 72°C for 5 min. The results of the PCR amplifications were checked on agarose gels. PCR products were cleaned with the Qiagen PCR Purification Kit and subsequently sequenced on an ABI 3100-Avant sequencer using the BigDye Terminator Cycle Sequencing Kit (Applied Biosystems). All sequences were deposited at GenBank (for accession numbers see Additional file 1).

### Phylogenetic tree reconstruction

Sequences were edited and aligned using BioEdit v7.5.0.2 [89], and manually checked by eye. For further analyses, identical sequences were removed. To exclude contaminations of the data set with pseudogenes, neighbor-joining trees based on uncorrected distances were reconstructed in PAUP* v4.0b10 [90] for both data sets separately, and the depicted relationships and branch lengths compared with each other. For all Bayesian and ML analyses, both data sets were combined and outgroup rooted with *Theropithecus gelada*. After the exclusion of a 3 bp-deletion in the ND5 gene of southern chacma baboons, the final alignment comprised 2,033 bp. Optimal nucleotide substitution models for each locus were chosen using AIC as implemented in MODELTEST v3.7 [91]. All ML analyses were conducted using a genetic algorithm approach implemented in GARLI v0.951 [92]. In GARLI only the model specifications settings were adjusted according to the data set; all other settings were left at their default value. Ten replicates were run to verify consistency in log likelihood (lnL) scores and tree topologies. ML bootstrap percentages (BP) were estimated in GARLI by performing 500 pseudoreplicate runs. PAUP* v4.0b10 [90] was then used to calculate a majority-rule consensus tree in order to obtain bootstrap percentages. Bayesian analyses were conducted on the concatenated data set using MrBayes v3.1.2 [93,94]. A partitioned analysis was performed treating the cytb and 'Brown region' as separate partitions, each with their own DNA substitution models. We used four Monte Carlo Markov Chains (MCMC) with the default temperature of 0.1. Four repetitions were run for ten million generations with tree and parameter sampling occurring every 100 generations. Flat priors were assumed for the model parameters including the proportion of invariable sites and the gamma shape parameter of rate variation among sites. The first 25% of samples were discarded as burnin, leaving 75,001 trees per run. The adequacy of this burnin and convergence of all parameters were assessed by examining the uncorrected potential scale reduction factor (PSRF) [95] as calculated by MrBayes v3.1.2 [93,94], which should approach 1 as runs converge and by visual inspection of the trace of the parameters across generations using the software TRACER v1.3 [96]. Posterior probabilities (PP) for each split and a phylogram with mean branch lengths were calculated from the posterior density of trees using MrBayes v3.1.2 [93,94]. Phylogenetic trees were visualized using TreeEdit v1.0a10 [97] and FigTree v1.1 [98]. For the description of the tree topology we defined clade as a group of at least three haplotypes with a common ancestor. For single terminal haplotypes or terminal haplogroups consisting of only two haplotypes we employed the term lineage.

### Estimation of divergence time

A Bayesian MCMC method, which employs a relaxed molecular clock approach [99], as implemented in BEAST v1.5beta2 [100], was used to estimate divergence times. A relaxed lognormal model of lineage variation and a Yule prior for branching rates was assumed. For the calculation we used the combined cytb and 'Brown region' sequences. To reduce the computational burden during analyses we included a subset of 18 specimens, because individuals either shared identical mitochondrial haplotypes or were relatively weakly differentiated. Nevertheless, we secured that the data set comprised representative sequences of all haplogroups. Additionally, we included our *Theropithecus gelada *sequence and 8 orthologous sequences from other primate taxa obtained from GenBank (see Additional file 1). After removing additional indels, which were present in outgroup taxa, the final alignment for divergence age estimations comprised 2,023 bp. The data set was partitioned into coding vs non-coding regions. The coding regions were then further partitioned according to 1+2 and 3 codon positions and the substitution model, rate heterogeneity and base frequencies were unlinked across codon positions ((1+2), 3). An optimal nucleotide substitution model was chosen using AIC as implemented in MODELTEST 3.7 [91].

As calibrations we used the divergence between human and chimpanzee, which has been dated at 6–7 mya [[101,102] in [103]], the divergence between orang-utan and African ape lineages, which is estimated at 14 mya [[104] in [105]], and the spilt between macaques and baboons, which is conservatively estimated at 7–8 mya [[106] in [103]].

Instead of hardbounded calibration points, we used the published dates as a normal distribution prior for the respective node (calibration points C1 and C2 are not shown in Figure 4). For C1 (*Pan*/*Homo*) this translates into a normal distribution with a mean of 6.5 mya and a standard deviation of 0.3 mya, for C2 (Ponginae/Homininae) into a mean of 14.0 mya and a standard deviation of 0.6 mya (95% credibility interval: 13.0–15.0 mya) and for C3 (Figure 4) into a mean of 7.5 mya and a standard deviation of 0.55 mya. Since the estimation of a tree topology was not the main aim of the analysis, we placed a monophyly constraint on the northern and southern *Papio *clade, respectively, thus attaining the same general topology as assessed by the full phylogenetic analyses.

Two replicates were run for 25 million generations with tree and parameter sampling occurring every 1,000 generations. The adequacy of a 10% burnin and convergence of all parameters were assessed by visual inspection of the trace of the parameters across generations using the softwares TRACER v1.4.1 [96] and AWTY [107]. Subsequently, the sampling distributions of two independent replicates were combined using the software LogCombiner v1.5beta2 and the resulting 45,002 samples summarized and visualized using the software TreeAnnotator v1.5beta2 and FigTree v1.2 [98]. The first two programs are part of the BEAST package [100].

## Authors' contributions

DZ conceived and coordinated the study, did most of the sampling, analyzed data and wrote the paper. LFG calculated phylogenies and divergence times, and wrote the paper. CK did sequencing and wrote the paper. CR did sequencing, analyzed data and wrote the paper. All authors read and approved the final manuscript.

## Supplementary Material

Additional File 1 **Geographic origin of samples and GenBank accession numbers**. In this table we provide information on the geographic origin of our samples, their haplotype designation, and the respective GenBank accession numbers for complete cytochrome b and 'Brown region' sequences. Click here for file
