# Correction to: Mitochondrial phylogeography of baboons (Papio spp.) – Indication for introgressive hybridization?

**DOI:** 10.1186/s12862-019-1537-6

**Published:** 2019-11-04

**Authors:** Dietmar Zinner, Linn F. Groeneveld, Christina Keller, Christian Roos

**Affiliations:** 1 Cognitive Ethology, Deutsches Primatenzentrum, Kellnerweg 4, D-37077 Göttingen, Germany; 2 Behavioral Ecology and Sociobiology, Deutsches Primatenzentrum, Kellnerweg 4, D-37077 Göttingen, Germany; 3 Institute of Farm Animal Genetics, Friedrich-Loeffler-Institut, Neustadt, Germany; 4 Göttinger Zentrum für Biodiversitätsforschung und Ökologie, Untere Karspüle 2, D-37073 Göttingen, Germany; 5 Gene Bank of Primates and Primate Genetics, Deutsches Primatenzentrum, Kellnerweg 4, D-37077 Göttingen, Germany

**Correction to: BMC Evol Biol (2009) 9:83**

**https://doi.org/10.1186/1471-2148-9-83**

Following publication of the original article [1], we have been notified that some of the NCB accession numbers were incorrectly associated to their corresponding taxon in the Additional file 1.

In this correction, the revised supplementary material is included.

## Supplementary information

**Additional file 1.** Geographic origin of samples and GenBank accession numbers. In this table we provide information on the geographic origin of our samples, their haplotype designation, and the respective GenBank accession numbers for complete cytochrome b and ‘Brown region’ sequences.
