# Supplementary material for: Mitochondrial phylogeography of baboons (Papio spp.) – Indication for introgressive hybridization?
Source: BMC Evol Biol. 2009 Apr 23;9:83. doi: 10.1186/1471-2148-9-83 (PMC2681462; doi:10.1186/1471-2148-9-83)
Supplement: Additional File 1 — Geographic origin of samples and GenBank accession numbers. In this table we provide information on the geographic origin of our samples, their haplotype designation, and the respective GenBank accession numbers for complete cytochrome b and 'Brown region' sequences. [file 1471-2148-9-83-S1.pdf]

Additional file 1: Sample localities (geographic coordinates in decimal degrees), haplotype designations and GenBank accession numbers for cytb and 'Brown region' haplotypes. Sequences used for divergence times estimates are marked by an asterisk.

id	taxon	country	site (side code)	longitude, latitude	haplotype	acc no cytb	acc no 'Brown'
096*	*Papio anubis*	Côte d'Ivoire	Comoe NP (KoN)	-3.79000 8.80000	A01	EU885420	EU885767
545	*P. anubis*	Nigeria	Bwari, Abuja (Bwa)	7.22030 8.81140	A02	EU885421	EU885768
546	*P. anubis*	Nigeria	Lumma, Niger State (Lum)	4.26420 10.31810	A03	EU885422	EU885769
550*	*P. anubis*	Nigeria	Lumma, Niger State (Lum)	4.26420 10.31810	A04	EU885423	EU885770
547*	*P. anubis*	Nigeria	Kemainja, Kwara State (Kem)	3.91670 9.71670	A05	EU885424	EU885771
548	*P. anubis*	Nigeria	Sepeteri, Oyo State (Sep)	3.65000 8.63330	A06	EU885425	EU885772
EY01	*P. anubis*	Nigeria	Gashaka Gumpti NP, Kwano (Kwa)	11.58333 7.31667	A06	EU885426	EU885773
EY10	*P. anubis*	Nigeria	Gashaka Gumpti NP, Kwano (Kwa)	11.58333 7.31667	A06	EU885427	EU885774
549*	*P. anubis*	Nigeria	Chigwa, Kaduna State (Chi)	7.81670 9.35000	A07	EU885428	EU885775
555	*P. anubis*	Nigeria	Chigwa, Kaduna State (Chi)	7.81670 9.35000	A07	EU885429	EU885776
552*	*P. anubis*	Nigeria	Kura, Plateau State (Kur)	9.26667 9.91667	A08	EU885430	EU885777
559	*P. anubis*	Cameroon	Primate Rescue Center Limbe (Lib)	13.93333 10.56667	A09	EU885431	EU885778
448*	*P. anubis*	Uganda	Kibale Forest (Kib)	30.40000 0.48333	A10	EU885432	EU885779
512	*P. anubis*	DRC	south Bukavu (sBu)	28.91092 -2.68258	A11	EU885433	EU885780
184	*P. anubis*	Eritrea	Ruba Griset (Gri)	36.76018 14.88322	A12	EU885434	EU885781
194	*P. anubis*	Eritrea	Ruba Hadejemi (Had)	36.90710 14.35827	A13	EU885435	EU885782
349	*P. anubis*	Ethiopia	Managasha NP (Man)	38.57125 8.96838	A14	EU885436	EU885783

527	*P. anubis*	Kenya	Segera Ranch (Seg)	36.01581 0.25338	A15	EU885437	EU885784
536	*P. anubis*	Kenya	Segera Ranch (Seg)	36.01581 0.25338	A16	EU885438	EU885785
353*	*P. anubis*	Tanzania	Serengeti NP (Swr)	34.85236 -2.43100	A17	EU885439	EU885786
507§	*P. cynocephalus*	Somalia	Webi Shebelli (Web)	45.43333 2.42083	C01	EU885440	EU885787
529*	*P. cynocephalus*	Kenya	Diani Beach (Dia)	39.55000 -4.32000	C02	EU885441	EU885788
533	*P. cynocephalus*	Kenya	Diani Beach (Dia)	39.55000 -4.32000	C02	EU885442	EU885789
537	*P. cynocephalus*	Kenya	Amboseli NP (Amb)	37.39000 -2.29000	C03	EU885443	EU885790
404§	*P. cynocephalus*	Tanzania	Lake Rukwa (Ruk)	32.15517 -7.58297	C04	EU885444	EU885791
151*	*P. cynocephalus*	Malawi	Michiru Mts. Cons. Area (Mic)	34.91667 -15.75000	C05	EU885445	EU885792
288	*P. cynocephalus*	Malawi	Mulanje Mt. (Mu2)	35.51667 -15.95000	C06	EU885446	EU885793
409	*P. cynocephalus*	Zambia	South Luangwa NP (LuS)	31.63793 -13.26840	C07	EU885447	EU885794
411	*P. cynocephalus*	Zambia	South Luangwa NP (LuS)	31.63793 -13.26840	C08	EU885448	EU885795
570	*P. cynocephalus*	Zambia	Luambe NP (Lua)	32.14550 -12.45780	C09	EU885449	EU885796
565*	*P. cynocephalus*	Zambia	Kafue NP, North (KfN)	26.53577 -14.96779	C10	EU885450	EU885797
568	*P. cynocephalus*	Zambia	Kasanka NP (Kas)	30.25202 -12.59059	C11	EU885451	EU885798
569*	*P. cynocephalus*	Zambia	Shiwa N`gandu (Shi)	31.73892 -11.19677	C12	EU885452	EU885799
037	*P. hamadryas*	Eritrea	Abdur (Abd)	39.84585 15.12857	H01	EU885453	EU885800
074	*P. hamadryas*	Eritrea	Kubkub (Kub)	38.63217 16.34482	H02	EU885454	EU885801
391	*P. hamadryas*	Eritrea	Afabet (Afb)	38.74958 16.12017	H03	EU885455	EU885802
301	*P. hamadryas*	Ethiopia	Awash Station (ASt)	40.17775 8.99268	H04	EU885456	EU885803
319	*P. hamadryas*	Ethiopia	Gerba Luku (Ger)	41.53400 9.58740	H05	EU885457	EU885804
414*	*P. hamadryas*	Yemen	Bura'a Forest, Hodaidah (BuH)	43.41667 14.86667	H06	EU885458	EU885805
526	*P. papio*	Senegal	Mt. Assirik, NK NP	-12.76667	P01	EU885459	EU885806

			(Ass)	12.88333			
556	*P. papio*	Senegal	Niokolo Koba, NK NP (Nio)	-12.72090 13.07467	P01	EU885460	EU885807
518	*P. papio*	Senegal	Kedougou (Ked)	-12.12472 12.57556	P02	EU885461	EU885808
523*	*P. papio*	Senegal	Kedougou (Ked)	-12.12472 12.57556	P03	EU885462	EU885809
252*	*P. papio*	Guinea	Bakaria, Haute Niger NP (Bak)	-10.31542 10.54267	P04	EU885463	EU885810
566	*P. ursinus*	Zambia	Kafue NP, Middle New Kalala (Nka)	26.01077 -15.77360	U01	EU885464	EU885811
567	*P. ursinus*	Zambia	Kafue NP, Middle (KfM)	25.99809 -15.79781	U01	EU885465	EU885812
501	*P. ursinus*	Zimbabwe	Nyanga (Nya)	32.81283 -19.05781	U02	EU885466	EU885813
539	*P. ursinus*	Mozambique	Gorongosa NP (Gor)	34.36111 -18.97833	U03	EU885467	EU885814
540	*P. ursinus*	Mozambique	Gorongosa NP (Gor)	34.36111 -18.97833	U03	EU885468	EU885815
492*	*P. ursinus*	Botswana	Moremi Wildlife Res (Mor)	23.00000 -19.18349	U04	EU885469	EU885816
422	*P. ursinus*	RSA	Pilanesberg Game Res (Pil)	26.87805 -25.11111	U05	EU885470	EU885817
425	*P. ursinus*	RSA	Pilanesberg Game Res (Pil)	26.87805 -25.11111	U05	EU885471	EU885818
478	*P. ursinus*	Namibia	Waterberg Plateau (Wat)	17.24221 -20.50450	U05	EU885472	EU885819
435	*P. ursinus*	RSA	Blyde River, Blydepoort (Bly)	30.78049 -24.66667	U06	EU885473	EU885820
563*	*P. ursinus*	RSA	Ithala Game Res (Ita)	31.26667 -27.53333	U07	EU885474	EU885821
564	*P. ursinus*	RSA	Ithala Game Res (Ita)	31.26667 -27.53333	U07	EU885475	EU885822
558	*P. ursinus*	Namibia	Okasewa Ranch (Oka)	18.34910 -22.41203	U08	EU885476	EU885823
470	*P. ursinus*	RSA	Loskop Nature Res (Los)	29.28162 -25.42147	U09	EU885477	EU885824
468	*P. ursinus*	RSA	Loskop Nature Res (Los)	29.28162 -25.42147	U10	EU885478	EU885825
560	*P. ursinus*	Angola	Serra Leba, Lubango (Lub)	13.24167 -15.14167	U11	EU885479	EU885826
472	*P. ursinus*	Namibia	Hakos Guestfarm (Hak)	16.36463 -23.23708	U12	EU885480	EU885827
482	*P. ursinus*	Namibia	Spreetshoogte NR (Spr)	16.20555 -23.64758	U12	EU885481	EU885828

541*	*P. ursinus*	RSA	Giant's Castle Game Res (Dra)	29.48333 -29.33333	U13	EU885482	EU885829
542	*P. ursinus*	RSA	Giant's Castle Game Res (Dra)	29.48333 -29.33333	U13	EU885483	EU885830
543	*P. ursinus*	RSA	Giant's Castle Game Res (Dra)	29.48333 -29.33333	U13	EU885484	EU885831
561	*P. ursinus*	RSA	Goegap Nature Res (Goe)	18.03222 -29.69944	U14	EU885485	EU885832
463*	*P. ursinus*	RSA	DeHoop Nature Res (Hop)	20.40658 -34.45621	U15	EU885486	EU885833
149	*Theropithecus gelada*		Zoo Duisburg, Germany		T01	EU885487	EU885834
divergence times estimates							
	Cebus albifrons					AJ309866	AJ309866
	Pongo pygmaeus					NC_001646	NC_001646
	Pan troglodytes					D38113	D38113
	Homo sapiens					AY339522	AY339522
	Colobus guereza					NC_00690	NC_00690
	Chlorocebus aethiops					NC_00700	NC_00700
	Macaca sylvanus					AJ309865	AJ309865
	M. mulatta					AY612638	AY612638

DRC = Democratic Republic of Congo; RSA = Republic of South Africa; [s]tissue from museum specimens
